# Targeted Therapies in Advanced Cholangiocarcinoma: A Focus on FGFR Inhibitors

**DOI:** 10.3390/medicina57050458

**Published:** 2021-05-08

**Authors:** Alessandro Rizzo

**Affiliations:** Department of Experimental, Diagnostic and Specialty Medicine, S. Orsola-Malpighi University Hospital, 40138 Bologna, Italy; rizzo.alessandro179@gmail.com

**Keywords:** FGFR, cholangiocarcinoma, targeted therapies, intrahepatic cholangiocarcinoma, pemigatinib

## Abstract

Despite advanced diseases continuing to be associated with grim prognoses, the past decade has witnessed the advent of several novel treatment options for cholangiocarcinoma (CCA) patients. In fact, CCA has emerged as a heterogeneous group of malignancies harboring potentially druggable mutations in approximately 50% of cases, and thus, molecularly targeted therapies have been actively explored in this setting. Among these, fibroblast growth factor receptor (FGFR) inhibitors have reported important results, as witnessed by the FDA approval of pemigatinib in previously treated metastatic CCA patients harboring FGFR2 fusion or other rearrangements. Herein, we provide an overview of available evidence on FGFR inhibitors in CCA, especially focusing on the development, pitfalls and challenges of emerging treatments in this setting.

## 1. Introduction

Cholangiocarcinoma (CCA) encompasses a group of heterogeneous, rare and aggressive malignancies, including intrahepatic cholangiocarcinoma (iCCA) and extrahepatic cholangiocarcinoma (eCCA), with the latter further subclassified into perihilar (pCCA) and distal (dCCA) cholangiocarcinoma [1,2,3]. CCAs account for approximately 3% of all gastrointestinal cancers worldwide and 10–15% of all primary liver tumors [4,5,6]. As suggested by several studies, these subgroups of hepatobiliary tumors not only develop from different anatomical locations, but vary widely in terms of epidemiology, biology, prognosis, and etiology [7,8,9]. 

Although radical surgical resections with negative tumor margins is the standard of care for early stages, for resectable diseases, only a small proportion of CCA patients are eligible for curative surgery at the time of diagnosis [10,11,12]. Adjuvant treatments have been actively explored in this setting, with the aim of lowering recurrence rates and improving the survival of patients [11]. In particular, adjuvant capecitabine has been recently established as standard treatment following radical surgery; in fact, this agent has been suggested to improve survival, according to the results of the phase III BILCAP trial [12,13,14]. Although the BILCAP failed to meet its primary endpoints according to an intention-to-treat analysis, in the prespecified per-protocol analysis (adjusted by nodal status, disease grade and gender) a statistically significant benefit in terms of median overall survival (OS) was reported (53 months versus 36 months; Hazard Ratio [HR] 0.75, 95% Confidence Interval [CI], 0.58–0.97; *p* = 0.028) [15].

As regards metastatic disease, combination chemotherapy with cisplatin plus gemcitabine (CisGem) represents the reference treatment for previously untreated patients with advanced CCA, following the landmark results of the ABC-02 and BT22 clinical trials [16,17,18]. More recently, for metastatic CCA patients whose disease progresses on front-line CisGem chemotherapy, second-line modified oxaliplatin plus 5-fluorouracil (mFOLFOX) plus active symptom control (ASC) has provided a survival benefit compared to ASC alone, according to the ABC-06 phase III trial [19,20]. However, the overall benefit provided by mFOLFOX is modest (median OS of 6.2 months in the ASC plus mFOLFOX group versus 5.3 months in the ASC alone group), and the overall response rate remains disappointing.

**Figure 1:**
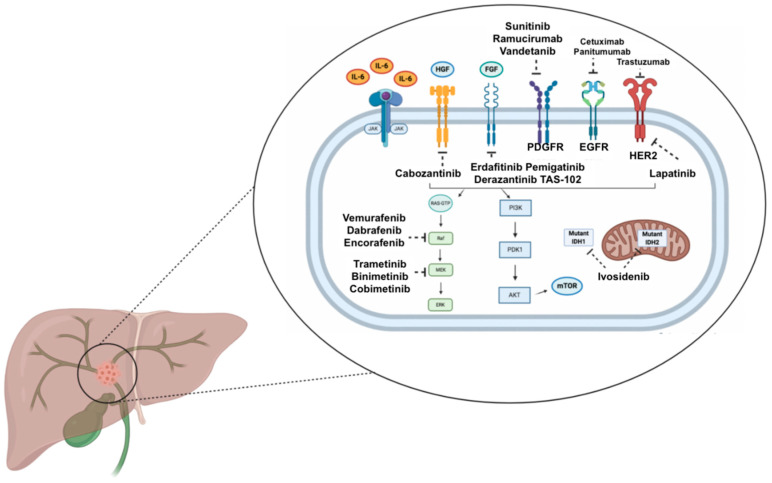
Schematic figure representing the main signaling pathways and selected targeted therapies currently under evaluation in cholangiocarcinoma. Abbreviations: AKT: protein kinase B; EGFR: epidermal growth factor receptor; FGF: fibroblast growth factor; HER2: epidermal growth factor receptor 2; HGF: hepatocyte growth factor; IL-6: interleukin 6; IDH: isocitrate dehydrogenase; JAK: Janus kinase; mTOR: mammalian target of rapamycin; PDGFR: platelet derived growth factor receptor; PDK1: phosphoinositide-dependent kinase-1; PI3K: phosphoinositide 3-kinase.

In fact, the overall limited survival benefit provided by systemic therapies in this setting, with most patients reporting a survival rate of less than a year from the moment of diagnosis, has led to notable efforts towards the identification of novel targets and agents that could modify the natural history of these aggressive hepatobiliary malignancies [20,21,22,23,24]. In fact, the massive use of next-generation sequencing (NGS) has led to the identification of previously unknown molecular features of CCA, including the presence of specific genetic aberrations that have been suggested to be distinctive features of iCCA and eCCA [25,26,27,28]. Among these druggable alterations, fibroblast growth factor receptor (FGFR)2 gene fusions and rearrangements, isocitrate dehydrogenase-1 (IDH-1) mutations, and BRAF mutations have been widely described in CCA patients, reporting important differences between iCCA and eCCA (Figure 1) [29,30,31,32].

In particular, FGFR-targeted treatments have entered into the clinical practice of CCA patients, since these agents have reported promising results in a number of phase I and II clinical studies [33,34,35]. In fact, in April 2020, the US Food and Drug Administration (FDA) granted accelerated approval of the FGFR inhibitor pemigatinib, on the basis of the results of the phase II FIGHT-202 trial—as we shall see later in more detail [36]. Moreover, several other FGFR inhibitors are being tested, together with studies aimed at better identifying mechanisms involved in secondary resistance [37,38,39,40,41,42]. 

Herein, we provide an overview of current evidence on FGFR inhibitors in CCA patients, especially focusing on the development of these molecules, as well as future research avenues in this setting. We performed research on PubMed/Medline, Cochrane library, and Scopus using the keywords “cholangiocarcinoma”, “intrahepatic cholangiocarcinoma”, “extrahepatic cholangiocarcinoma”, “biliary tract cancer”, “FGFR”, “FGFR2”, “pemigatinib”, “derazantinib”, “infigratinib”, “erdafitinib”, and “futibatinib”. We selected pivotal registration studies. We also selected the most relevant and pertinent studies considering the quality of the studies in terms of their applicability, how they were conducted, statistical analysis, number of patients enrolled, and outcomes. For ongoing clinical trials, we searched in the clinicaltrials.gov database for recruiting and active, not recruiting trials, using the following keywords: “cholangiocarcinoma”, “intrahepatic cholangiocarcinoma”, “extrahepatic cholangiocarcinoma”, “biliary tract cancer”, “FGFR”, “FGFR2”, “pemigatinib”, “derazantinib”, “infigratinib”, “erdafitinib”, and “futibatinib”. We restricted our research to phase one, two, or three trials.

## 2. FGFR Aberrations in Cholangiocarcinoma

**Figure 2:**
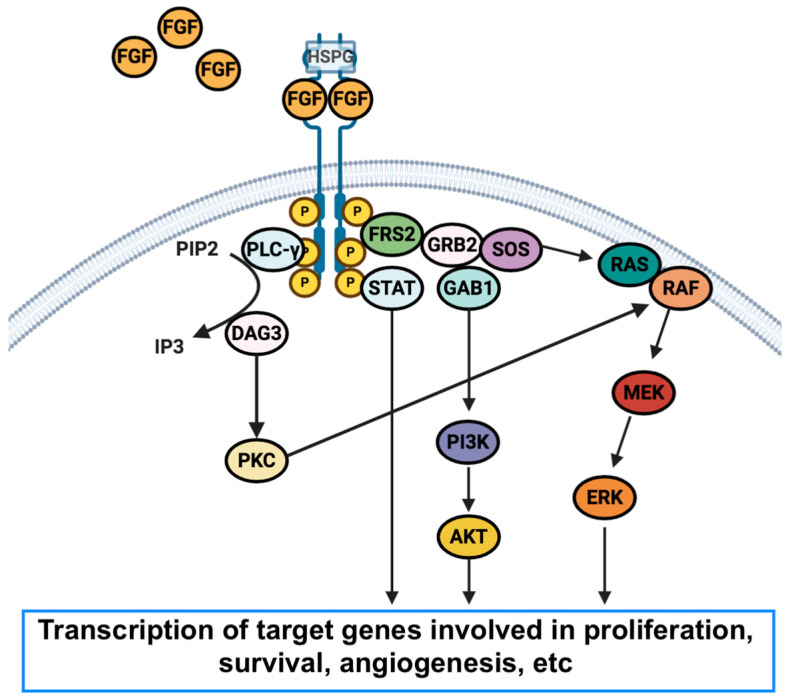
Schematic figure reporting the structure of the Fibroblast Growth Factor Receptor (FGFR), the network, and alteration in tumors. Abbreviations: FRS2: fibroblast growth factor receptor substrate 2; HSPG: heparan sulfate proteoglycan; PLC-γ: phospholipase gamma; PIP2: phosphatidylinositol 4,5-bisphosphate; IP3: phosphatidylinositol 3,4,5-triphosphate; DAG: diacylglycerol; PKC: protein kinase C; GRB2: growth factor receptor-bound protein 2; GAB1: GRB2-associated-binding protein; MEK: MAPK/ERK Kinase.

The FGFR receptors family consists of five different receptors: FGFR1, FGFR2, FGFR3, FGFR4, and FGFR5 [43]; while the first four receptors present tyrosine kinase domains, FGFR5 does not, and thus, the fifth receptor does not seem to be involved in carcinogenetic processes [44]. Notably enough, FGFR-related signaling plays a crucial role in modulating angiogenesis, differentiation, intracellular survival and cell proliferation, and genetic aberrations in FGFRs have been highlighted in several malignancies [45]. In particular, the interaction between FGFRs and their ligands hesitates in the dimerization of the receptor, with the transphosphorylation of the tyrosine kinase domains [46,47]. This process results in the activation of a number of pathways, including JAK/STAT, phospholipase Cγ (PLCγ), RAS-dependent mitogen-activated protein kinase (MAPK), and phosphatidylinositol 3-kinase (PI3KCA)/Akt/mTOR [45,46,47] (Figure 2).

FGFR aberrations have reported a variable frequency in different malignancies, with urothelial carcinoma and iCCA having been observed as the most common [48]. As regards specific aberrations in FGFRs, these events have mainly been highlighted in gene encoding for FGFR2, particularly in terms of gene rearrangements or fusions, while amplifications and/or mutations are considered rarer [49,50,51]. Moreover, several FGFR1-4 aberrations have been observed across different malignancies, and FGFR/FGF alterations are detected in approximately 7% of all solid tumors, according to a landmark study conducted by Helsten and colleagues. In this report, gene amplifications appeared to be the most frequent alteration (66%), followed by mutations (26%). Notably enough, FGFR dysregulations are involved in oncogenic signaling, by inducing aberrant expression of FGFRs or enhanced activity—in the case of mutations of FGFR overexpression. In the specific setting of CCA, from their first identification, FGFR2 gene fusions have been suggested to represent a unique clinical and molecular subtype of iCCA, that has been reported in approximately one fifth of all iCCAs [52,53]. In fact, patients with FGFR2 gene fusions are frequently female subjects, younger than wild-type CCAs, and usually present a less aggressive clinical course [54]; in addition, these gene fusions seem to be mutually exclusive with BRAF and KRAS mutations and have been observed almost uniquely in iCCAs. Conversely, FGFR aberrations are considered an anecdotal finding in dCCAs, eCCAs, and other biliary tract cancers, including gallbladder cancers and ampulla of Vater tumors [55]. The first evidence of FGFR2 fusions in iCCA patients was reported by Wu and colleagues in 2013 [53]; subsequently, an impressive number of studies have been published on this topic, also observing that etiology and geographical elements could modify the prevalence of FGFR aberrations in CCA. Recent years have registered the advent of several different methods able to identify FGFR2 fusions, including traditional immunohistochemistry, polymerase chain reaction (PCR), fluorescent in situ hybridization (FISH), and other approaches based on NGS [56]. Nonetheless, these methods are not superimposable since they have been associated with considerable differences in terms of comparability, reproducibility and specificity. 

## 3. FGFR-Targeted Therapies in CCA: Non-Selective and Selective Inhibitors

Over the last decade, several clinical studies have evaluated the role of FGFR-directed therapies. In particular, several attempts have been made in order to develop targeted treatments in this setting, including the use of small molecule inhibitors (such as ATP competitive small molecule FGFR inhibitors and covalent small molecule inhibitors), recombinant peptides, monoclonal antibodies, and antibody drug conjugates [57]. Early studies on FGFR inhibition in CCA patients were mainly focused on non-selective inhibitors, such as lenvatinib, pazopanib, regorafenib, and dovitinib [58,59,60,61]. In particular, following case reports and case series showing the promising activity of the non-selective tyrosine kinase inhibitors ponatinib and pazopanib, these agents have been tested in preclinical and clinical trials. However, several studies have shown important issues associated with the use of non-selective FGFR tyrosine kinase inhibitors, including short-term responses and disappointing clinical outcomes. More recently, the CCA medical community has focused its attention on the development of specific and selective FGFR inhibitors, including infigratinib, derazantinib, erdafitinib, pemigatinib, futibatinib, and debio 1347 [62]. Most of these compounds share several features, including the reversible bind to a highly conserved P-loop cysteine residue in an ATP pocket—with the important exception of futibatinib, as we shall see later. 

### 3.1. Infigratinib

The FGFR1, FGFR2, and FGFR3 selective tyrosine kinase inhibitor infigratinib (BJG398) has represented the first FGFR inhibitor reporting promising results in CCA clinical trials [63]. Firstly, a dose-escalation and dose-expansion study tested this molecule in 132 patients with advanced malignancies harboring FGFR genetic aberrations (NCT01004224); according to the results of this study, the recommended phase 2 dose for the FGFR inhibitor was 125 mg once daily (3 weeks on, 1 week off schedule) [64]. The final results of a phase II trial conducted by Javle and colleagues have been recently presented at the 2021 Gastrointestinal Cancers Symposium organized by the American Society of Clinical Oncology (ASCO) (NCT02150967) [65,66]. According to the updated findings of this single-arm, multicenter study in 108 pretreated advanced CCA with FGFR2 fusion or rearrangement, infigratinib showed an overall response rate (ORR) of 23.1%, with a median duration of response of 5.0 months and median PFS of 7.3 months [65]. In terms of treatment-related adverse events, the most frequently observed toxicities were hyperphosphatemia, fatigue, stomatitis, and alopecia, with grade 3–4 treatment-related adverse events as hyponatremia and hypo- or hyperphosphatemia [65,66].

### 3.2. Derazantinib

Another FGFR-targeted agent, derazantinib (ARQ087), has been tested in CCA patients in recent years [67]; this molecule is a pan-FGFR inhibitor also able to inhibit several other kinases, such as RET, VEGFR1, DDR, and KIT [68]. Firstly, a phase I study including 80 patients with advanced malignancies defined 300 mg once daily as the recommended phase II dose (NCT01752920) [68]. Subsequently, the role of derazantinib in 29 iCCAs harboring FGFR2 fusion was evaluated in a phase I/II, open-label trial conducted by Mazzaferro and colleagues (NCT01752920) [69]. In this study including previously treated patients or patients not eligible for front-line chemotherapy CCA, derazantinib (300 mg once daily) reported a disease control rate (DCR) and ORR of 82.8% and 20.7%, respectively [69]. Based on these results, derazantinib is being assessed in the phase II FIDES-01 trial evaluating the FGFR inhibitor in pretreated iCCA patients (NCT03230318); in this study, one arm includes patients with FGFR2 gene fusions, while the second cohort presents patients with FGFR2 mutations or amplifications. 

### 3.3. Erdafitinib

The potent tyrosine kinase inhibitor erdafitinib has shown activity against all four FGFRs, also reporting efficacy against several other kinases [70]. Despite this agent having reported notable results in urothelial carcinoma, few data are available regarding the role of erdafitinib in CCA patients with FGFR alterations [71,72]. In particular, a phase IIa, open-label trial conducted on 17 Asian patients with previously treated CCA harboring FGFR alterations (10 FGFR2 fusions, four FGFR2 mutations, one FGFR3 fusion, and two FGFR3 mutations) observed a partial response (PR) and stable disease (SD) in 46.7% and 33.3% of cases, respectively [73,74]. According to the results of this study, ORR was 47% and DCR 80% in the 15 CCA patients with an evaluable response. Erdafitinib was administered at the dosage of 8 mg once daily, on 28-day cycles; in addition, the study design allowed to increase the dose to 9 mg once daily in the absence of significant erdafitinib-related hyperphosphatemia [74].

### 3.4. Pemigatinib

The FGFR1, FGFR2, FGFR3 inhibitor pemigatinib represents the FGFR-directed agent at the most advanced stage of development, since this molecule has represented the first targeted agent to be approved in CCA so far (Table 1) [75]. Firstly, the phase I/II FIGHT-101 trial evaluated pemigatinib in patients with previously treated solid tumors with or without FGFR aberrations (NCT02393248) [76]. Notably enough, the dose-escalation part of this study established 13.5 mg once daily as the recommended phase II dose for pemigatinib (on days 1 to 14 of each 21-day cycle). Based on these premises, the open-label, multicenter, FIGHT-202 trial tested pemigatinib in pretreated CCA patients harboring FGFR2 gene fusions or rearrangements (*n* = 107), other FGFR aberrations (*n* = 20), or without FGFR aberrations (*n* = 18) (NCT02924376) [77]. At a median follow-up of 17.8 months, the 35% (38/107) of patients with FGFR2 fusions or rearrangements showed an objective response, including three cases of complete responses. Conversely, the authors reported no responses in the other two groups of patients harboring other FGFR aberrations or without mutations [77,78]. In terms of survival outcomes, the FGFR inhibitor reported notable results in patients with FGFR2 fusions or other rearrangements, with a median PFS and median OS of 6.9 months and 21.1 months, respectively. On the contrary, disappointing outcomes were observed in the other two cohorts—a median OS of 2.1 months and 1.7 months in CCA patients harboring other FGFR aberrations or without mutations. Lastly, in terms of safety profile, pemigatinib-related adverse events mirrored the toxicities observed with infigratinib and derazantinib, since hyperphosphatemia was the most frequently observed all-grade adverse event, reported in 60% of included patients. Moreover, 64% of the subjects experienced grade three or four toxicities, the most frequent of which were hypophosphatemia (12%) and arthralgia (6%). These results have led to the US FDA approval of pemigatinib for previously treated patients with advanced or metastatic disease harboring FGFR2 fusion or other rearrangements identified by the FoundationOne^®^ CDX (Foundation Medicine, Inc., Cambridge, MA, USA) test [79].

### 3.5. Debio 1347

Another molecule, the highly selective FGFR1, FGFR2, and FGFR3 inhibitor Debio 1347, is being tested in solid tumors harboring FGFR aberrations, including CCA [80]. Debio 1347 was firstly evaluated in a first-in-human trial on advanced solid tumors, reporting a safety profile acceptable up to 80 mg daily (NCT01948297) [80]; this study evaluated Debio 1347 in 18 patients, including five cases of CCA (four patients with FGFR2 fusion and one case of FGFR1 fusion). According to the results of this early-phase study, in patients with FGFR2 fusion, SD was reported in two cases, as well as PR in two patients; conversely, the patient with FGFR1 fusion experienced a progressive disease [81]. Based on these premises, the phase II, multicenter, open-label FUZE (NCT03834220) trial aimed to investigate Debio 1347 in pretreated malignancies with FGFR fusions, including CCAs, urothelial carcinomas, and other solid tumors [82]. At the time of writing, the FUZE has completed enrolment and results of this trial are being awaited (Table 2).

### 3.6. Futibatinib

The highly selective, irreversible FGFR1, FGFR2, FGFR3, and FGFR4 inhibitor futibatinib (TAS-120) was firstly evaluated in a phase I study, the FOENIX-101, where 86 patients with previously treated advanced malignancies received this molecule (NCT02052778) [42,83,84,85]. According to the results of this dose-escalation trial, 20 mg once daily was established as the recommended phase II dose, with PR reported in five patients and SD in 41 subjects [83,84,85]. Based on these preliminary findings, futibatinib was assessed in the phase II FOENIX-CCA2 trial evaluating the FGFR inhibitor in CCA patients who had experienced disease progression on standard treatments or were not eligible for standard therapy [42,86]. Notably enough, the early results of the FOENIX-CCA2 phase II trial have been presented at the ESMO World Congress on Gastrointestinal Cancer 2020; futibatinib monotherapy reported an ORR and a DCR of 34.3% and 76.1%, respectively, in 67 CCA patients with FGFR2 fusions or other rearrangements [42,86]. In recent years, the CCA medical community has shown growing attention towards this molecule, since several studies have observed that futibatinib could be active in CCA patients pretreated with other FGFR inhibitors, suggesting a possible role in overcoming acquired resistance due to the irreversible binding of this molecule [42,87].

## 4. Open Questions and Future Research Avenues

Despite FGFR inhibitors having entered into clinical practice for CCA, several questions remain unanswered, including the presence of resistance to targeted therapy that may be present at the start of the treatment or form over time. In fact, as in the case of several treatments in other oncogene-addicted malignancies, primary and secondary resistance represent important issues in CCA patients treated with FGFR-targeted treatments [88,89,90]. With regard to the former, a report by Silverman and colleagues has recently observed a trend towards worse clinical outcomes in CCAs reporting specific FGFR fusion partners, including BAP1, PBRM1, CDKN2A/B, and TP53. However, few data are available on this topic [91]. Conversely, mechanisms involved in acquired resistance have been studied more extensively in the last few years.

Firstly, a landmark study by Goyal and colleagues observed the first piece of evidence of secondary resistance to FGFR-directed treatment in three CCA patients harboring FGFR2 fusion [92]; all these patients were treated with infigratinib, and all three patients reported the FGFR2 V565F gate-keeper mutation. In addition, two patients also showed polyclonal secondary mutations in the FGFR2 kinase domain [92]. Notably enough, the authors conducted this study by using integrative genomic characterization of cell-free circulating tumor DNA (cfDNA). In particular, serial analysis of cfDNA reported multiple recurrent point mutations at progression, and these findings were mirrored by the biopsy of post-progression lesions and autopsy. Based on these premises, the duration of response for FGFR-targeted treatments seems limited by the onset of multiple FGFR2 mutations in the kinase domain. More recently, Goyal et al. published a proof-of-concept study on four CCA patients with FGFR2 fusion treated with infigratinib or Debio 1347 [93]. At disease progression, patients received futibatinib that bound covalently to FGFR, as previously reported; according to the results of this study, two patients experienced PR, staying on futibatinib for 16 and 17 months, respectively [93]. Thus, futibatinib has the potential to play an important future role in determining the best treatment sequence in this setting. Despite there still being a long time until liquid biopsy can be introduced in this setting in everyday clinical practice, cfDNA and circulating tumor DNA are being evaluated in CCA patients, and ongoing clinical trials will provide further information on this emerging and important topic in CCA management.

In addition, with the aim of improving therapeutic options and extending survival in CCA patients, an impressive number of clinical trials is evaluating the use of FGFR inhibitors in patients with FGFR2 fusion or rearrangement. In particular, a generation of studies is trying to establish whether FGFR inhibitors could overcome CisGem in treatment-naïve patients harboring druggable mutations. Among these, the phase III PROOF trial (NCT03773302) for infigratinib, and the phase III FIGHT-302 (NCT03656536) trial for pemigatinib are currently recruiting patients; conversely, a similar study with futibatinib, the FOENIX-CCA3 (NCT04093362) trial, is preparing to open for enrolment. In addition, another series of studies is evaluating combination strategies, including FGFR inhibitors plus other anticancer agents (e.g., systemic chemotherapy, immunotherapy) in solid tumors with FGFR fusions, including CCA [94,95,96].

Lastly, another recent research avenue is the study of the intensive cross-communication of FGFRs and other cancer-related proteins, that has been suggested to have the potential to constitute a therapeutic target for cancer patients. In particular, several studies have highlighted the cross-talk of FGFR and other proteins implicated in solid tumors, such as galectins [97,98,99,100]. Although this evidence is still preliminary, this strategy has attracted much attention by the CCA medical community.

## 5. Conclusions

The recent approval of the FGFR inhibitor pemigatinib in previously treated patients with advanced CCA harboring FGFR2 gene fusions or rearrangements has heralded a new era in CCA treatment. Nonetheless, a plethora of challenges remains to be overcome, including the challenges associated with the development of drug resistance [101,102]. Many ongoing clinical trials are evaluating the efficacy of FGFR inhibitors in the front-line setting, both as a single agent and in combination with other anticancer agents, that might help to improve the outcome of patients with advanced CCA. Identifying targetable genomic alterations through liquid biopsy is another exciting frontier for future exploration.

## Figures and Tables

**Table 1:** Main characteristics of pemigatinib in terms of route of administration, pharmacokinetics and pharmacodynamics.

Drug Names	Pemigatinib; IBI-375; INCB-054828; INCB-54828; Pemazyre
Molecular formula	C_24_H_27_F_2_N_5_O_4_
Chemical name	3-(2,6-difluoro-3,5-dimethoxyphenyl)-1-ethyl-8-(morpholin-4-ylmethyl)-1,3,4,7-tetrahydro-2*H*-pyrrolo [3′,2′:5,6]pyrido[4,3-d]pyrimidin-2-one
Route of administration	13.5 mg once daily, orally, on days 1-14 of a 21-day cycle
Pharmacokinestics	Proportional increase of concentrations over a 1-20 mg dose range at steady state; median time to maximum plasma pemigatinib concentration is 1.13 h
Pharmacodynamics	Selective inhibitor of FGFR1, FGFR2, and FGFR3
Most common toxicities	Hyperphosphatemia, alopecia, diarrhea, fatigue, dysgeusia

**Table 2:** Ongoing clinical trials on FGFR inhibitors in advanced cholangiocarcinoma.

Agent	NCT number	Phase	Patient Population
Infigratinib versus Gemcitabine Cisplatin	NCT03773302	III	Advanced Cholangiocarcinoma
Infigratinib	NCT04233567	II	Advanced, Metastatic, or Refractory Malignant Solid Neoplasm
Derazantinib	NCT03230318	II	Intrahepatic Cholangiocarcinoma Combined Hepatocellular and Cholangiocarcinoma
Derazantinib	NCT04087876	Expanded Access	Intrahepatic Cholangiocarcinoma
Erdafitinib	NCT02699606	IIa	Advanced, Metastatic, or Refractory Malignant Solid Neoplasm
Erdafitinib	NCT03210714	II	Advanced, Metastatic, or Refractory Malignant Solid Neoplasm
Erdafitinib	NCT04083976	II	Advanced, Metastatic, or Refractory Malignant Solid Neoplasm
Erdafitinib	NCT02465060	II	Advanced, Metastatic, or Refractory Malignant Solid Neoplasm
Ponatinib	NCT02272998	II	Advanced, Metastatic, or Refractory Malignant Solid Neoplasm
Ponatinib	NCT02265341	II	Advanced, Metastatic, or Refractory Hepatobiliary Malignancy
Futibatinib versus Gemcitabine Cisplatin	NCT04093362	III	Advanced Cholangiocarcinoma with FGFR2 Gene Rearrangements
Futibatinib	NCT04507503	Expanded Access	Advanced Cholangiocarcinoma
Futibatinib	NCT04189445	II	Advanced, Metastatic, or Refractory Malignant Solid Neoplasm
Debio 1347	NCT03834220	II	Advanced, Metastatic, or Refractory Malignant Solid Neoplasm
Pemigatinib	NCT04003623	II	Advanced, Metastatic, or Refractory Malignant Solid Neoplasm
Pemigatinib	NCT03822117	II	Advanced, Metastatic, or Refractory Malignant Solid Neoplasm
Pemigatinib versus Gemcitabine Cisplatin	NCT03656536	III	Advanced Cholangiocarcinoma
Pemigatinib	NCT04256980	II	Advanced Cholangiocarcinoma
Pemigatinib	NCT04258527	I	Advanced, Metastatic, or Refractory Malignant Solid Neoplasm
Gemcitabine Cisplatin plus ivosidenib or pemigatinib	NCT04088188	I	Advanced Cholangiocarcinoma

## Data Availability

Not applicable.
